# Unlocking the potential of blockchain technology in enhancing the fisheries supply chain: an exploration of critical adoption barriers in China

**DOI:** 10.1038/s41598-024-59167-4

**Published:** 2024-05-03

**Authors:** Ubair Nisar, Zhixin Zhang, Bronwyn P. Wood, Shadab Ahmad, Ehsan Ellahi, Syed Ijaz Ul Haq, Mohamad Alnafissa, Elsayed Fathi Abd-Allah

**Affiliations:** 1 School of Economics, Shandong University of Technology, Zibo, Shandong People’s Republic of China; 2 College of Business and Economics, United Arab Emirates University, Al Ain, UAE; 3 School of Mechanical Engineering, Shandong University of Technology, Zibo, China; 4 College of Agricultural Engineering and Food Science, Shandong University of Technology, Zibo, China; 5 Department of Agricultural Economics, College of Food and Agricultural Sciences, King Saud University, P.O. Box. 2460, 11451 Riyadh, Saudi Arabia; 6 Plant Production Department, College of Food and Agricultural Sciences, King Saud University, P.O. Box. 2460, 11451 Riyadh, Saudi Arabia

**Keywords:** Blockchain, Supply chain sustainability, Critical barrier, DEMATEL, China, Ocean sciences, Mathematics and computing

## Abstract

The application of blockchain technology holds significant potential for improving efficiency, resilience, and transparency within the Fisheries Supply Chain (FSC). This study addresses the critical barriers hindering the adoption of blockchain technology (BT) in the Chinese FSC, recognizing the unique challenges posed by its intricacies. Through a comprehensive literature review, fourteen Critical Barrier Factors (CBFs) were identified, and a grey Delphi method was employed to distill this set. Five pivotal CBFs emerged, including "Regulatory Compliance," "Cost of Implementation," and "Complex Supply Chain Network". A subsequent grey Decision-Making Trial and Evaluation Laboratory (DEMATEL) analysis revealed the causal relationships among these factors, categorizing them into effect and cause groups. "Regulatory Compliance," "Cost of Implementation," and "Complex Supply Chain Network" were identified as primary influencing factors demanding attention for effective BT integration in the FSC. The findings serve as a valuable resource for FSC stakeholders, assisting in prioritizing efforts to address these barriers. The discerned causal relationships provide guidance for managers in optimizing resource allocation. Ultimately, this research advocates for the adoption of blockchain technology in the fisheries supply chain to enhance overall performance and operational efficiency.

## Introduction

The Chinese fisheries supply chain plays a pivotal role in both fulfilling China's domestic seafood consumption needs and facilitating its active participation in the global seafood trade. As one of the world's foremost producers and consumers of seafood, China's fisheries supply chain operates as a multifaceted and ever-evolving network. It involves a diverse array of stakeholders, ranging from fishermen and aquaculture farmers to processors, distributors, and exporters. China's fisheries sector encompasses a vast spectrum of aquatic products, encompassing a diverse range of fish species, shellfish, and aquatic plants. The current major crisis China is facing is ever-increasing seafood demand, water scarcity, and cultivable land resources. In order to overcome these problems, the best solution is intensive sustainable aquaculture and efficient supply chain management^1^. This comprehensive portfolio solidifies China's position as a key player in the worldwide seafood industry. The significance of this supply chain extends not solely to meeting the demands of China's vast population but also to its substantial exports of seafood products to international markets. China as a nation reduces the impact of future uncertainties by improving its supply chain management and logistics networking^2^.

Blockchain technology has garnered significant attention in recent years, drawing interest from both researchers and professionals across various industries^3^. Blockchain, as a decentralized peer-to-peer platform, presents a particularly promising prospect for implementation within complex food supply chains. The blockchain technology model named Deep Improving Commute Experience (DeepICE) demonstrate the superior performance of proposed model compared to existing approaches^4^. Unlike many other industries, the food production sector operates within intricate value chains that necessitate heightened attention to handling and storage. The utilization of blockchain technology has the potential to greatly enhance the trustworthiness, effectiveness, and protection of data shared among participants in supply chain networks^5^. Factors such as transportation and temperature can significantly impact the quality and freshness of food products^6^. The primary factor that positions Blockchain as the pivotal tool of our times lies in its distinctive attributes. These include the instantaneous exchange of information, robust cybersecurity, transparency, dependability, traceability, and enhanced visibility, all of which contribute to the optimization of supply chain operations^7^. In the fisheries industry, blockchain technology can be leveraged to record data from specialized IoT devices, such as intelligent sensors. These sensors capture vital information from the moment of product capture all the way through to the final delivery to the end customer. The data they provide, particularly regarding factors like temperature, is crucial for monitoring the condition of transported products. Beyond the utilization of blockchain technology, there is a requisite for additional adaptable sensors. These sensors not only facilitate precise and accurate detection of crucial changes in environmental parameters but also offer promising resolutions for various challenges, including enhanced precision in agri-food processing, grading, and inspection^8^. Blockchain technology can play a vital role in ensuring the integrity of transportation, handling, and storage processes, including tamper-proof checks and maintaining a comprehensive product history, among other applications^9^. Leveraging effective blockchain adoption (BCA) alongside enhanced knowledge management (KM) within organizational and production processes has emerged as a potent combination for elevating sustainable organizational performance (SOP)^10^.

In today's rapidly changing global landscape, supply chains (SCs) encounter numerous challenges. One major issue is the difficulty in accurately forecasting demand due to historical demand data being unreliable^11,12^. Additionally, inadequate communication among various players within the supply chain leads to limited visibility and an increased risk of unexpected problems arising^13^. The modern supply chain is often lengthy and intricate, which can result in the bullwhip effect caused by poor communication between different entities. This negatively impacts overall supply chain operations, emphasizing the necessity to rethink how information is utilized for more effective and efficient supply chain coordination and performance improvement^14^. The fast-paced nature of today's business environment adds further complexity to supply chain collaboration^15^, especially when considering the diversity of social and organizational cultures. End-to-end supply chains encompass multi-tier supply chains (MTSCs), involving the collection and analysis of vast amounts of data. This demands a transparent workflow for sharing information across all levels of the MTSC. Consequently, collaboration becomes a crucial component in addressing the current challenges faced by supply chains. Furthermore, this technology has the potential to alleviate various supply chain challenges, including issues related to data loss, transparency, accuracy, and dependable communication. Blockchain technology is viewed as a valuable tool for rebuilding trust among supply chain partners by providing a secure and credible platform for sharing information. As a result, blockchain technology is considered a significant emerging trend that is poised to have a substantial impact on both business and society in the years ahead^16^. Lately, sustainability has taken the forefront as the primary research focus, encompassing aspects such as compliance with environmental protection regulations, the integration of eco-friendly technologies, regulatory obligations, and the implementation of agile supply chain strategies^17^.

Following the advent of Industry 4.0, emerging economies are currently undergoing a progressive shift in technology adoption^18^. This transformation can be effectively orchestrated through the implementation of Industry 3.5 strategies, as opposed to strict adherence to the lofty objectives of Industry 4.0. Industry 3.5 represents a balanced approach that integrates elements from both Industry 4.0 and Industry 3.0, leveraging recent advancements in information and communication technologies^19^. Although there is increasing enthusiasm for Blockchain Innovation (BI), there is a scarcity of research concerning its determinants^20^. Blockchain technology is strategically positioned to pave the way for innovative business models and has the potential to spearhead such advancements^21^. Traditionally, blockchain is described as a secure ledger of historical transactions, organized into blocks, arranged chronologically, and distributed across multiple servers to establish a streamlined provenance^22^. It facilitates peer-to-peer transactions and offers significant enhancements in terms of transparency, accountability, security, efficiency, and cost reduction^23^. Additionally, blockchain technology guarantees data immutability, traceability, and the implementation of smart contracts, fostering high-trust environments without the need for intermediaries^24^. In the realm of technological research, scholars have primarily concentrated on the significance of fostering technological innovation within the supply industry for economic development^25^.

Furthermore, the blockchain-based system eliminates the need for intermediaries from both public and private institutions, resulting in a significant reduction in transaction costs. Participants in the blockchain-based system place their trust in computer code rather than relying solely on themselves, ensuring a highly secure and foolproof process^26^. Blockchain technology introduces traceability into the FSC while smart contracts facilitate seamless operations within the agri-business sector. Additionally, the blockchain-based system enhances the sustainability of supply chain operations by meticulously tracking compliance for each activity^27,28^. Although blockchain technology holds great promise for revolutionizing FSC, its practical adoption is still in its nascent stages^21,29^. Much like technology-focused nations, China is keen on harnessing the potential of blockchain to meet its growing demands and requirements. Therefore, there is a need to investigate the current barriers to blockchain adoption. Consequently, the objectives of this research can be succinctly summarized as follows:

1. Identify the barriers to blockchain adoption in the Chinese Fisheries Supply Chain (FSC).
2. Develop a model to analyze how these barriers relate to each other and establish a hierarchy.
3. Assess the strength of causal relationships among the identified factors and categorize them accordingly.

The primary aim of the study is to identify the barriers hindering the adoption of blockchain technology (BT) in the FSC and to discover the cause-and-effect relationships between these barriers. The Goal of the study is to provide valuable insights that can assist decision-makers at government policy and company levels in taking effective actions to address these obstacles and promote the successful implementation of BT in the sector. While some prior research has indeed identified barriers to BT adoption, the study goes a step further by establishing the causal connections between these barriers, thereby offering practical guidance to practitioners on where to concentrate their efforts when implementing blockchain. To achieve the research objectives, we conducted a comprehensive literature review to identify the most significant barriers to adopting BT in FSC. Recognizing these significant barriers is crucial for facilitating BT adoption in this context. However, the obstacles identified are substantial and cannot all be tackled simultaneously. Therefore, to ensure the successful integration of BT in supply chains, the study recognized the need to construct a causal relationship map. This map serves as a systematic approach to address these barriers in a prioritized manner. Furthermore, when organizations understand the cause-and-effect relationships among these apparent barriers, they can allocate their resources more efficiently in mitigating these challenges. One distinctive contribution of our study, particularly relevant to the context of a developing economy like China, is the identification of this causal structure of BT implementation barriers in the suppl chain of fisheries sector.

## Literature review

### Blockchain and supply chain

A supply chain is a highly intricate, adaptable network that spans various stages, geographical locations, financial systems, and entities. The dynamics of a supply chain vary based on product type and market conditions^30,31^. Supply chain systems encompass networks of facilities and distribution entities, including suppliers, manufacturers, distributors, and retailers. These systems execute the functions of procuring raw materials, transforming them into intermediates and finished products, and distributing the final products to customers. This process is achieved through the control of both information flow and material flow^32^. These networks typically involve multiple partners, such as manufacturing facilities, distribution centers, suppliers, logistics providers, and couriers^33^, contributing to their growing complexity. Several factors have contributed to this complexity, including sustainability concerns, globalization, trade liberalization, reduced trade costs, and the integration of new technologies. Effective management of supply chain networks is critical for maintaining organizational competitiveness^30,34^. It is imperative to advance technological solutions aimed at addressing challenges within the cold chain concerning perishable commodities^35^.

In recent times, blockchain technology has garnered significant attention from both researchers and practitioners for its potential role within supply chains. It offers various benefits, including the use of smart contracts, product traceability, enforcement tracking, inventory management, transaction and settlement facilitation, and data immutability^36^. To attain optimal efficiency in the product flow within a supply chain, it is imperative to pioneer the development of novel technologies that can mitigate losses^37^. Existing literature has identified numerous applications of blockchain within supply chain management. An extensive literature review focusing on blockchain-based applications within the Agricultural Supply Chain (ASC)^21^. Their research findings shed light on the various uses of blockchain in ASC, encompassing traceability, sustainable water management, agri-food manufacturing, and information security. A survey was conducted to gauge the landscape of blockchain research in agriculture^38^. They observed that the adoption of blockchain in this sector is still in its early stages. The authors categorized existing blockchain-based research into four dimensions: traceability, architecture, information systems, and other miscellaneous applications. In a related context^39^, advocated the incorporation of blockchain technology for ensuring food safety, particularly when coupled with Radio-Frequency Identification (RFID) technology. Subsequently, in 2017, Tian proposed a traceability mechanism for the Agricultural Supply Chain (ASC) by harnessing the combined power of blockchain and the Internet of Things (IoT). Additionally, Yadav and Singh introduced a framework designed to address specific challenges faced by farmers in the Indian context. They proposed the use of a blockchain-based mobile application as a solution to these challenges^40^.

Existing studies in the SC perspective regarding blockchain applications are categorized into four major types: "conceptual", "descriptive", "predictive," and "prescriptive" research. For instance^41^, studied BT and identified the potential areas of BT contribution to performance from a SC perspective. Further, they also highlighted the scope for future research, from which the motivation for this study was derived, of highlighting on BT barriers and their interrelationships. Reference^42^ proposed blockchain based system architecture and found that BT can decrease the complexity of the management of SC. A literature review was conducted review and pinpointed privacy and security as the central hurdles in the implementation of blockchain technology^43^. Lu conducted a review focusing on blockchain technology (BT), identifying its essential components, its role in data management, security enhancement, BT-based Internet of Things (IoT), and primary applications^44^. Furthermore, Lu discussed emerging trends and the associated challenges in the field of BT. A review of blockchain technology was carried out, emphasizing the potential benefits of immutable distributed ledgers in supply chain operations^28^. Finally,^45^ explored challenges related to blockchain-enabled IoT and investigated how blockchain technology can enhance the performance of the Internet of Things.

A study was conducted on blockchain trends within the Agricultural Supply Chain (ASC) and explored the associated challenges hindering its widespread adoption. These challenges encompass factors such as the absence of government regulation, uncertainties regarding regulatory frameworks, inadequate training resources, and related issues^46^. Kamble^47^ delved into the subject of blockchain-based traceability mechanisms for the food-retail Supply Chain (SC) in India. Simultaneously, undertook an analysis of the hurdles encountered when applying blockchain to food traceability^48^. One notable challenge lies in the lack of control over sensors that feed data into the blockchain system. This makes it challenging to detect fraudulent manipulations of such sensors. The adoption of blockchain in ASC is further complicated by a range of other obstacles, including the shortage of skilled professionals, regulatory gaps, limited system storage capacity, throughput and latency issues, scalability concerns, privacy considerations, and the associated high costs^21^. The concept of using blockchain for land registration in the Indian context, emphasizing the potential benefits of authenticity and tamper-proof record-keeping was explored^49^. However, they also acknowledged various implementation challenges, including the substantial initial investment required, regulatory uncertainties, and security-related concerns.

### Blockchain fisheries adoption

The rapid pace of technological innovation has made the swift adoption of information communication technologies a critical objective for businesses. The fisheries industry is notably complex due to the multitude of products, processes, individuals, and organizations it encompasses^50^. For instance, Ireland's fishing sector comprises distinct segments like refrigerated seawater pelagic, beam trawler, polyvalent, specific, and aquaculture, each focusing on different types of aquatic species and products. In Ireland, aquaculture primarily revolves around salmon farming, and fish processing involves numerous companies, with many generating revenues exceeding €1 million. The industry predominantly consists of whitefish, pelagic, and shellfish operators, with whitefish, shellfish, and smoked salmon processors being particularly prominent. This intricate structure is a consequence of the globalization, distribution, and consumption patterns in the food production sector^50,51^. To tackle the intricacies of supply chain management in this context, various solutions have been proposed, including vigilant information systems and blockchain technology^9^.

Numerous theories have emerged over time to explain the factors driving the adoption of information technology. These theories often examine user behavior, such as the Technology Acceptance Model (TAM), Task-Technology Fit (TTF) theory, Diffusion of Innovation (DOI) theory, Theory of Reasoned Action (TRA), Theory of Planned Behavior (TPB), Unified Theory of Acceptance and Use of Technology (UTAUT), and Social Cognitive Theory (SCT)^36^. Other models include the Perceived e-Readiness Model and Assimilation Theory^52^. In 1990, the Technology-Organization-Environment (TOE) model was introduced, identifying three distinct areas within an organization's context that influence the adoption and implementation of technological innovations: the technological context, organizational context, and environmental context^53^. The technological context encompasses both internal and external technologies relevant to the firm, including factors like complexity, relative advantage, privacy, security, and compatibility, all of which have been shown to impact information technology adoption^36,53^. The organizational context considers the firm's scope, size, managerial structure, top management support, prior IT experience, innovativeness, information intensity, and organizational readiness^53^.

### Applications

Our research contributes by systematically identifying and categorizing fourteen specific Critical Barrier Factors (CBFs) through an extensive literature review and the use of the grey Delphi method. This establishes a structured foundation for understanding the hurdles that impede the successful integration of blockchain technology in fisheries supply chains. The work will provide valuable insights into the challenges associated with the adoption of blockchain technology in emerging economies within the fisheries industry. By highlighting the multifaceted nature of these challenges, including logistical complexities, financial constraints, and regulatory intricacies, the study contributes to a nuanced understanding of the specific hurdles faced by these regions. The study also delves into the intricate challenges posed by regulatory compliance in the seafood industry. By recommending strategies such as open communication, integration of compliance features into blockchain design, and leveraging smart contracts, the article contributes practical insights for overcoming regulatory hurdles in the adoption of blockchain technology. Recognizing the unique socio-economic and regulatory environment of China, the study identifies specific barriers that can hinder the seamless integration of blockchain across the entire supply chain. This contextual understanding contributes to the development of tailored strategies for overcoming challenges in this specific market.

## Methodology

**Figure 1:**
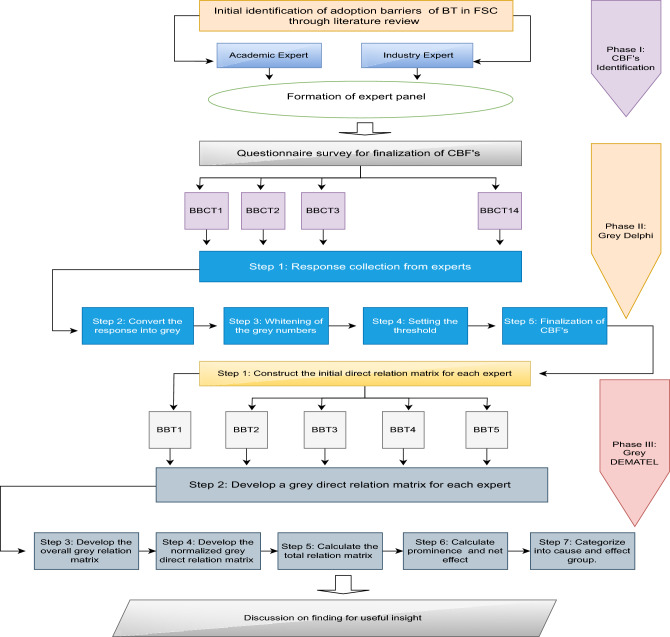
Proposed framework for CBFs of blockchain adoption in FSC.

This study follows a three-phase research framework to accomplish its stated research objectives as shown in Fig. 1. *Initial CBF Identification*: the first phase commences with a systematic literature review aimed at identifying the preliminary Critical Barrier Factors (CBFs) associated with the Blockchain-based FSC. To achieve this, a comprehensive review of existing literature is conducted using the Scopus database. Scopus is chosen due to its recognition as one of the largest repositories of peer-reviewed articles in the fields of science and social sciences. Through this systematic review, the initial set of CBFs for blockchain adoption within the FSC is established. *Expert Involvement and CBF Finalization*: the second phase involves the utilization of the grey Delphi method to finalize the identified CBFs for blockchain adoption within the FSC. Expert input plays a pivotal role in this phase, facilitating the refinement and selection of the most relevant CBFs. This expert-driven approach ensures a comprehensive and informed understanding of the critical factors. *Causal Relationship Development*: subsequent to the finalization of the CBFs for blockchain adoption, the third phase employs the grey Decision-Making Trial and Evaluation Laboratory (DEMATEL) technique to construct causal interrelationships among these factors. Various techniques, including Interpretive Structural Modelling (ISM), Total Interpretive Structural Modelling (TISM), Analytic Network Process (ANP), and DEMATEL, have been utilized in the literature to explore causal relationships^54^. While ISM and TISM reveal structural linkages between CBFs, they do not quantify the strength of these relationship^16^. The DEMATEL approach, however, provides a means to measure the strength of each relationship. To address potential subjectivity and vagueness in expert input, this study combines grey theory with DEMATEL, aiming to enhance the precision and reliability of the causal relationships among the CBFs^55^.

Grey Delphi and Grey DEMATEL are particularly well-suited for handling situations with uncertain and incomplete information. In the context of our study on the adoption barriers of blockchain technology in the Chinese Fisheries Supply Chain, the presence of uncertainties warranted a methodology that could effectively manage and process imperfect information. Grey systems theory provides a flexible framework that allows for the incorporation of uncertainties in decision-making processes. Given the dynamic and complex nature of the barriers within the supply chain, we found Grey Delphi and Grey DEMATEL to be suitable for modeling and evaluating the interactions among critical barrier factors. The choice of Grey Delphi and Grey DEMATEL has been successfully applied in prior studies involving complex decision-making scenarios. This includes situations where there is a need to evaluate and prioritize factors within supply chains, making them relevant and applicable to our investigation in the Chinese Fisheries Supply Chain. Grey systems theory allows for a more straightforward interpretation of results, which is essential for conveying findings to a diverse audience, including stakeholders and practitioners in the fisheries industry. This aligns with our objective to ensure that the outcomes of our study are accessible and actionable.

The data collection protocol for this research is a methodically structured process aimed at acquiring high-quality input from a diverse group of experts in supply chain management and blockchain technology. The initial step involved a careful selection of experts, considering variations in professional backgrounds, experiences, and educational qualifications to ensure a comprehensive perspective. After identifying potential participants, informed consent was obtained, outlining the purpose, confidentiality, and voluntary nature of their involvement. To gather relevant and insightful information, tailored questionnaires were designed for the Grey Delphi and Decision-Making Trial and Evaluation Laboratory (DEMATEL) methodologies. These questionnaires are crafted to elicit detailed responses on critical barrier factors affecting the adoption of blockchain technology in the Chinese Fisheries Supply Chain. Descriptive data, including the designation, work experience, country of residence, educational background, and gender of each expert, was collected to provide context and potentially identify patterns within the expert panel. Furthermore, the participants' history of engagement in Grey Delphi, DEMATEL, or both was recorded, allowing for an understanding of their familiarity with the methodologies. Similarly, the distribution of electronic questionnaires is conducted systematically, with periodic reminders to ensure timely and complete responses. In cases where additional clarification or insights are needed, follow-up interviews were conducted in a structured manner. Data validation is a crucial step to ensure the accuracy and reliability of the collected information. Cross-referencing responses and addressing any discrepancies or outliers enhances the integrity of the dataset. Subsequently, the collected data undergoes rigorous analysis, involving Grey Delphi iterations and DEMATEL analysis. These methodologies are employed to quantify qualitative responses and construct a matrix of pairwise comparisons, facilitating a robust examination of critical barrier factors in the adoption of blockchain technology within the Chinese Fisheries Supply Chain. Finally, the aggregated findings were presented in a clear and comprehensive manner, adhering to scientific standards. This meticulous data collection protocol ensures the credibility and validity of the research outcomes, contributing to a nuanced understanding of the complexities surrounding blockchain technology adoption in the fisheries supply chain context. The expert details are provided in Table 1.

**Table 1:** Descriptive data about expert panelists.

S. no	Designation	Work experience in years	Country	Education	Gender	Participated in grey Delphi	Participated in DEMATEL
1	Professor	28	China	Doctorate	Male	Yes	Yes
2	Professor	38	China	Doctorate	Female	Yes	Yes
3	Professor	17	China	Doctorate	Male	Yes	No
4	Supply Chain manager	18	China	Masters	Male	Yes	No
5	Blockchain Technologist	7	China	Masters	Female	Yes	Yes
6	Financial Analyst	31	China	Masters	Male	Yes	Yes
7	Supply Chain Manager	19	China	Masters	Male	Yes	No
8	Logistics Manager	12	China	Masters	Male	Yes	Yes
9	Blockchain Designer	8	China	Masters	Male	Yes	Yes

### Preliminaries

To address the issue of uncertainties inherent in expert input for Multiple Criteria Decision Making (MCDM) methods, this study incorporates the theory of grey systems^56^, a methodology widely used across various domains. Specifically, this research combines grey systems theory with Delphi and DEMATEL techniques to handle subjective data provided by expert panels.

In the grey systems framework, information is categorized into three distinct groups: fully certain information is denoted as white, inadequate information is represented as grey, and absolute unknown information is indicated as black^57^. In this study, the concept of interval grey numbers from the grey system theory has been applied. As a result, the essential definitions and operations of interval grey numbers are provided as follows:

#### Definition 1

Suppose $\otimes$ G implies the interval grey number that could be expressed as:

1 $$\otimes {\text{G}}=\left[\underset{\_}{{\text{G}}},\overline{{\text{G}} }\right]=\left[{{\text{G}}}^{{{\prime}}}\in {\text{G}}\mid \underset{\_}{{\text{G}}}\le {{\text{G}}}{{^{\prime}}}\le \overline{{\text{G}} }\right]$$

where, $\underset{\_}{{\text{G}}}$ shows the lower limit and $\overline{{\text{G}} }$ depicts the upper limits of the information $\otimes$ G.

#### Definition 2

The fundamental mathematical operations on the interval grey numbers are provided as follows:

Addition of two interval grey number is expressed as Eq. (2):

2 $$\otimes {{\text{G}}}_{1}+\otimes {{\text{G}}}_{2}=\left[{\underset{\_}{{\text{G}}}}_{1}+{\underset{\_}{{\text{G}}}}_{2},{\overline{{\text{G}}} }_{1}+{\overline{{\text{G}}} }_{2}\right]$$

Subtraction of interval grey number is expressed as Eq. (3):

3 $$\otimes {{\text{G}}}_{1}-\otimes {{\text{G}}}_{2}=\left[{\underset{\_}{{\text{G}}}}_{1}-{\overline{{\text{G}}} }_{2},{\overline{{\text{G}}} }_{1}-{\underset{\_}{{\text{G}}}}_{2}\right]$$

Multiplication of interval grey number is expressed as Eq. (4):

4 $$\otimes {{\text{G}}}_{1}\times \otimes {{\text{G}}}_{2}=\left[{\text{min}}\left({\underset{\_}{{\text{G}}}}_{1}{\underset{\_}{{\text{G}}}}_{2},{\underset{\_}{{\text{G}}}}_{1}{\overline{{\text{G}}} }_{2},{\overline{{\text{G}}} }_{1}{\underset{\_}{{\text{G}}}}_{2},{\overline{{\text{G}}} }_{1}{\overline{{\text{G}}} }_{2}\right),{\text{max}}\left({\underset{\_}{{\text{G}}}}_{1}{\underset{\_}{{\text{G}}}}_{2},{\underset{\_}{{\text{G}}}}_{1}{\overline{{\text{G}}} }_{2},{\overline{{\text{G}}} }_{1}{\underset{\_}{{\text{G}}}}_{2},{\overline{{\text{G}}} }_{1}{\overline{{\text{G}}} }_{2}\right)\right]$$

Division of interval grey number is expressed as Eq. (5):

5 $$\otimes {{\text{G}}}_{1}\div \otimes {{\text{G}}}_{2}=\left[{\underset{\_}{{\text{G}}}}_{1},{\overline{{\text{G}}} }_{1}\right]\times \left[\frac{1}{{\underset{\_}{{\text{G}}}}_{2}},\frac{1}{{\overline{{\text{G}}} }_{2}}\right]0\notin \otimes {{\text{G}}}_{2}$$

#### Definition 3

Usually, the whitenisation value (crisp value) of the interval grey number $\otimes {G}_{i}=\left[\underline{G},\overline{G }\right]$ is done with $\otimes$ and could be done through Eq. (6).

6 $$\widetilde{\otimes }={\upalpha }\cdot \underset{\_}{{\text{G}}}+(1-{\upalpha })\cdot \overline{{\text{G}} },{\upalpha }=[\mathrm{0,1}].$$

where, a is the coefficient of whitenisation and the commonly used value for a is 0.5, considering the equal weight mean whitenisation.

### Grey Delphi

Dalkey and Helmer^58^ introduced the Delphi approach, a widely used survey methodology designed to aggregate expert opinions on a particular issue with the goal of achieving a collective consensus^59^. However, the traditional Delphi method has certain drawbacks, including the need for multiple rounds to reach a consensus, susceptibility to subjectivity, and potential expert biases. To address these limitations, the integration of grey theory is proposed^60^. Consequently, the combination of Delphi and grey set theories results in the creation of the grey Delphi approach. The following outlines the key steps involved in implementing the grey Delphi method:

*Step 1: identifying the obstacles to blockchain adoption within the Fisheries Supply Chain (FSC).*

This stage encompasses the recognition of possible adoption hindrances within the FSC by conducting a thorough literature review. Utilizing these identified Critical Barrier Factors (CBFs), a questionnaire is then crafted to gather expert input and data.

*Step 2: Response compilation from experts.*

Gathering responses from experts involves distributing the constructed questionnaire to these individuals, who are requested to furnish their answers using a linguistic scale. The linguistic scales and corresponding grey numbers for each scale can be found in Table 2.

**Table 2:** Linguistic terms and their corresponding grey scales.

Linguistic scale	Very low important (VL)	Low important (LI)	Medium important (M)	High important (H)	Very high important (VH)
Grey number	(0,1)	(1,2)	(2,3)	(3,4)	(4,5)

*Step 3: Comprehensive evaluation using grey number.*

Comprehensive assessment employing grey numbers is carried out by converting the responses into corresponding grey numbers. These grey numbers serve as the basis for consolidating the feedback provided by the panel of experts, which, in this context, consists of k members. The evaluation of the factor $\otimes {{\text{G}}}_{{\text{i}}}$ is as follows:

7 $$\otimes {{\text{G}}}_{{\text{i}}}=\frac{\left(\otimes {{\text{G}}}_{{\text{i}}}^{1}+\otimes {{\text{G}}}_{{\text{i}}}^{2}+\dots +\otimes {{\text{G}}}_{{\text{i}}}^{{\text{h}}}+\dots +\otimes {{\text{G}}}_{{\text{i}}}^{{\text{k}}}\right)}{{\text{k}}}$$

where $\otimes {{\text{G}}}_{{\text{i}}}$ i is the overall evaluation of adoption factors and $\otimes {{\text{G}}}_{{\text{i}}}^{{\text{h}}}$ denotes the hth expert’s evaluation of CSF ‘i’ of blockchain adoption barrier.

*Step 4: Whitening of the grey number.*

The grey number having the interval $\left(\otimes {{\text{G}}}_{{\text{i}}}=[\underset{\_}{{\text{G}}},\overline{{\text{G}} }]=\left[{{\text{G}}}^{{{\prime}}}\in {\text{G}}\mid \underset{\_}{{\text{G}}}\le {{\text{G}}}^{{{\prime}}}\le \underset{\_}{{\text{G}}}\right]\right)$ and their equivalent whitenisation value is $\widetilde{\otimes }$ . The whitenisation of grey numbers is obtained through Eq. (6) as mentioned in preliminary section.

*Step 5: Setting threshold limit and CBFs selection.*

**Figure 2:**
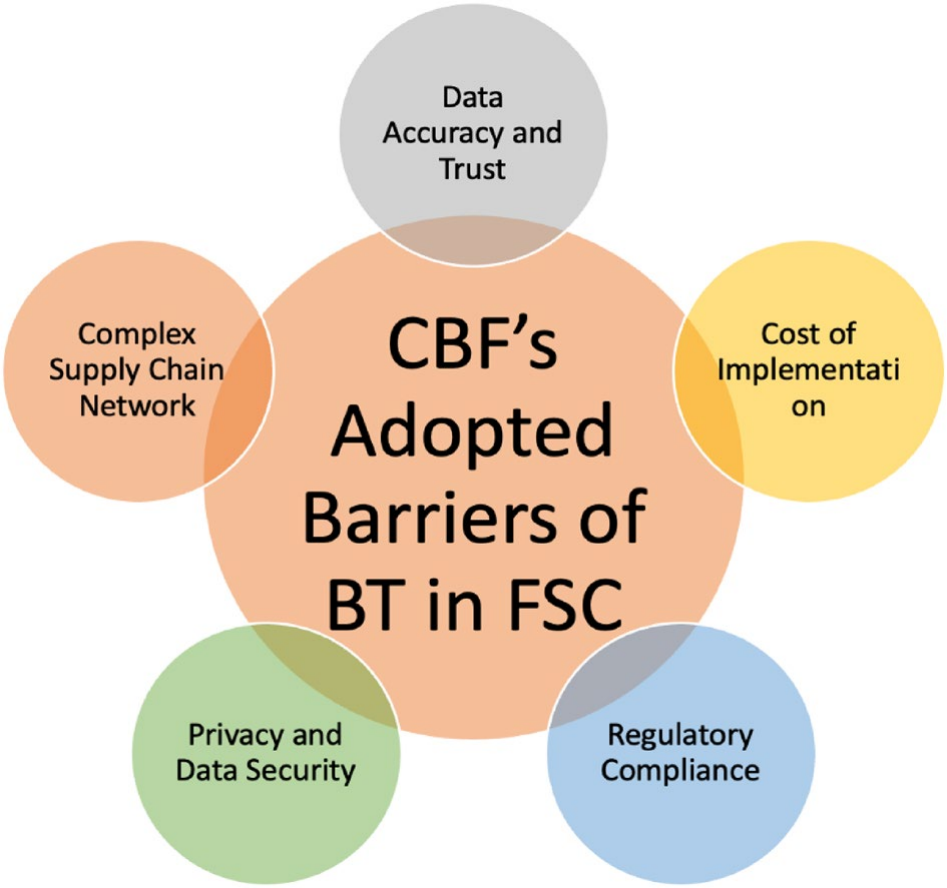
CBFs of BT adoption barriers in FSC.

The final step of the grey Delphi method involves selecting and rejecting CBFs. In order to determine the significance of the factor, an overall score is calculated and compared to a threshold value $(\uplambda )$. If the value of $\widetilde{\otimes }\ge\uplambda$, then the factor is selected; otherwise, it is rejected. The accepted CBF’s are shown in Fig. 2.

### Grey DEMATEL

A grey DEMATEL approach combines grey theory and DEMATEL to address the incomplete information, subjectivities, and biases in expert input. Furthermore, this method can improve the accuracy of the observations. Following is the stepwise procedure for grey DEMATEL.

*Step 1: Construct the initial direct-relation matrix.*

Develop an initial direct relation matrix of CBFs (c = {ci|i = 1, 2,, n}) of blockchain adoption barriers n FSC, utilizing the five-point grey linguistic scale (refer to Table 3), from “No Influence” to “Very High influence” by k experts through pair-wise comparisons.

**Table 3:** Linguistic scale for influential score.

Scale	Interpretation
(0,1)	Very low important (VL)
(1,2)	Low important (L)
(2,3)	Medium important (M)
(3,4)	High important (H)
(4,5)	Very high important (VH)

*Step 2: Develop a grey direct-relation matrix.*

The linguistic term is substituted with the corresponding grey number to convert the obtained initial direct relationship matrix into a grey initial direct relationship matrix. As K-number of experts provided their responses, the K grey direct relationship matrix X1, X2, X3…, XK is obtained. The direct-relation grey matrix is represented as follows by Eq. (8):

8 $${{\text{X}}}^{{\text{k}}}=\left[\begin{array}{cccccc}0& \otimes {{\text{x}}}_{12{\text{k}}}& \otimes {{\text{x}}}_{13{\text{k}}}& \cdots & \otimes {{\text{x}}}_{1({\text{n}}-1){\text{k}}}& \otimes {{\text{x}}}_{1{\text{nk}}}\\ \otimes {{\text{x}}}_{21{\text{k}}}& 0& \otimes {{\text{x}}}_{23{\text{k}}}& \cdots & \otimes {{\text{x}}}_{2({\text{n}}-1){\text{k}}}& \otimes {{\text{x}}}_{2{\text{nk}}}\\ \vdots & \vdots & \vdots & \ddots & \vdots & \vdots \\ \otimes {{\text{x}}}_{({\text{n}}-1)1{\text{k}}}& \otimes {{\text{x}}}_{({\text{n}}-1)2{\text{k}}}& \otimes {{\text{x}}}_{({\text{n}}-1)3{\text{k}}}& \cdots & 0& \otimes {{\text{x}}}_{({\text{n}}-1){\text{nk}}}\\ \otimes {{\text{x}}}_{{\text{n}}1{\text{k}}}& \otimes {{\text{x}}}_{{\text{n}}2{\text{k}}}& \otimes {{\text{x}}}_{{\text{n}}3{\text{k}}}& \cdots & \otimes {{\text{x}}}_{{\text{n}}({\text{n}}-1){\text{k}}}& 0\end{array}\right]$$

The element of [X], $\otimes {{\text{x}}}_{{\text{ijk}}}=\left(\underset{\_}{\otimes }{{\text{x}}}_{{\text{ijk}}},\overline{\otimes }{{\text{x}} }_{{\text{ijk}}}\right)$, shows the influence of CBF ‘i’ on CBF ‘j’ by the kth expert. The x ijk represents the lower and xijk the upper limit of grey values.

*Step 3: Develop the overall grey relation matrix.*

To formulate the overall grey relation matrix, all grey direct relation matrices were combined by using Eq. (9).

9 $$\otimes {\text{X}}=\sum_{{\text{i}}=0}^{{\text{K}}} \left(\frac{\sum \underset{\_}{\otimes }{{\text{x}}}_{{\text{ijk}}}}{{\text{K}}},\frac{\sum \overline{\otimes }{{\text{x}} }_{{\text{ijk}}}}{{\text{K}}}\right)$$

*Step 4: Express the normalized grey direct-relation matrix. In order to transform the grey relation matrix into a normalized grey direct-relation matrix N,*

Equations (4)–(6) are used.

10 $$\otimes \mathbf{s}=[\underset{\_}{{\text{s}}},\overline{{\text{s}}}]=\frac{1}{\underset{0\le {\text{i}}\le {\text{n}}}{{\text{max}}} \sum_{{\text{j}}=0}^{{\text{n}}} \otimes {{\text{X}}}_{{\text{ij}}}}{\text{i}},{\text{j}}=\mathrm{1,2},3\dots {\text{n}}.$$

11 $${\text{N}}=\otimes \mathbf{s}\cdot {\text{X}}$$

12 $$\otimes {{\text{n}}}_{{\text{ij}}}=\left[\underset{\_}{{\text{s}}}.\otimes {{\text{x}}}_{{\text{ij}}},\overline{{\text{s}}}.\otimes {{\text{x}}}_{{\text{ij}}}\right]$$

*Step 5: Calculate the total relation matrix.*

The total relation matrix “T” is obtained from the normalized grey direct-relation matrix by using Eq. (13).

13 $$\otimes {\text{T}}=\otimes {\text{N}}\cdot (\otimes {\text{I}}-\otimes {\text{N}}{)}^{-1}$$

where “I” is the grey identity matrix.

*Step 6: Compute the causal parameters.*

The Eqs. (14) and (15) are used to determine the causal parameter:

14 $$\otimes {\mathbf{R}}_{{\text{i}}}=\sum_{{\text{j}}=1}^{{\text{n}}} {{\text{t}}}_{{\text{ij}}}{\forall }_{{\text{i}}}$$

15 $$\begin{array}{c}\\ \otimes {\mathbf{C}}_{{\text{j}}}=\sum_{{\text{i}}=1}^{{\text{n}}} {{\text{t}}}_{{\text{ij}}}{\forall }_{\mathbf{j}}\end{array}$$

The R_i_ shows the influence of the CSF “i”, which infers the overall influence of CBFs, and the Cj represents the influence received by “j” by the other CBFs.

*Step 7: Calculate the prominence (Pi) and net effect (Ei)*

The prominence (Pi) and net effect (Ei) score of the CBFs is determined using expressions (16) and (17):

16 $$\otimes {\mathbf{P}}_{{\text{i}}}=\otimes {\mathbf{R}}_{{\text{i}}}+\otimes {\mathbf{C}}_{{\text{j}}}\mid {\text{i}}={\text{j}}$$

17 $$\begin{array}{c}\\ \otimes {{\text{E}}}_{{\text{i}}}=\otimes {{\text{R}}}_{{\text{i}}}-\otimes {{\text{C}}}_{{\text{j}}}\mid i=j\end{array}$$

The causal relationship diagram is constructed using the prominence (Pi) and net effect (Ei) scores. A positive Ei value indicates that a CBF causes an effect on the system, while a negative Ei value signifies its impact on the CBF itself.

## Results

### Identifying obstacles in implementing blockchain technology within the fisheries supply chain

The literature review uncovered several challenges hindering the effective implementation of blockchain technology (BT) in fisheries within the context of supply chains (SCs). To identify relevant scholarly articles, the study relied on the Scopus database, a renowned repository of scientific literature. A targeted search in the Scopus database using keywords such as 'fisheries supply chain management,' 'fish- supply chain' 'blockchain technology,' 'supply chain obstacles,' 'challenges,' and 'barriers were conducted. The search query is created by using Boolean operators to combine these keywords. Subsequently, these queries are executed in the Scopus database to locate relevant articles. Following this relevant academic works were selected based on an initial assessment of the article's abstract and title. Subsequently, an exhaustive examination of the existing literature, resulting in the identification of fourteen specific Critical Barrier Factors (CBFs) that impede the successful integration of blockchain technology into supply chains were conducted. These obstacles can be found in Table 4.

**Table 4:** Barriers in adoption of blockchain technology in fisheries supply chain.

S. no	Code	Barriers	References
1	BBCT1	Complex supply chain network	^61^
2	BBCT2	Data accuracy and trust	^62,63^
3	BBCT3	Data standardization	^31^
4	BBCT4	Integration with legacy systems	^64,65^
5	BBCT5	Cost of implementation	^66,67^
6	BBCT6	Resistance to change	^68^, expert opinion
7	BBCT7	Regulatory compliance	^69–71^
8	BBCT8	Scalability	^72,73^
9	BBCT9	Environmental concerns	^71,74^
10	BBCT10	Privacy and data security:	^21,49,61,75^
11	BBCT11	Lack of technical recourse	^76,77^
12	BBCT12	Lack of new organizational policies for using blockchain technology	^78,79^
13	BBCT13	The complexity of blockchain based system design	^61^
14	BBCT14	Supply chain fragmentation	^80,81^

After initially identifying the Critical Barrier Factors (CBFs) for the adoption of blockchain technology in FSC, the grey Delphi method with the assistance of experts were applied. To ensure a well-rounded perspective, experts from both academia and industry were selected^16^. The study assembled a group of professionals from the FSC and technology development sectors, each of whom possessed a minimum of five years of experience in FSC management. Additionally, the work enlisted academic experts specializing in FSC, technology management, or technology transfer, who were affiliated with respected academic institutions. The selection of these experts was based on their demonstrated expertise and knowledge in the field of FSC. As a result, a total of 9 valid responses were collected, which are presented in Table 5.

**Table 5:** Expert’s assessment of CBF to BT implications in FSC.

CBF	E1	E2	E3	E4	E5	E6	E7	E8	E9
BBCT1	M	H	H	M	VH	M	VH	H	VH
BBCT2	VH	VH	VH	VL	VH	VH	H	VH	H
BBCT3	VH	VH	VH	L	L	M	H	M	L
BBCT4	H	H	H	VH	H	L	H	M	H
BBCT5	H	H	VH	VH	H	M	H	H	VH
BBCT6	VH	M	H	VH	H	L	M	L	VL
BBCT7	H	H	VH	H	H	VH	H	VH	M
BBCT8	M	VH	H	M	M	VL	M	VL	L
BBCT9	H	VH	H	VL	VH	H	H	H	VL
BBCT10	VH	H	VH	VL	VH	VH	VH	VH	M
BBCT11	M	H	H	VH	H	M	VH	H	M
BBCT12	M	M	H	H	M	H	VH	H	VL
BBCT13	VH	H	VH	M	M	VH	H	VH	VL
BBCT14	M	M	VH	VH	M	H	VH	VL	H

Following the collection of feedback from the panel of experts, the transformation of the linguistic values into numerical representations was done using Table 2. The resulting grey matrix is displayed in Table 6. This table serves as the numerical foundation for the subsequent application of the Grey Delphi method, facilitating a systematic and quantitative analysis of expert opinions on the critical barriers influencing the adoption of blockchain technology in the Chinese Fisheries Supply Chain.

**Table 6:** Grey data as inputs to the grey Delphi method.

CBF	E1	E2	E3	E4	E5	E6	E7	E8	E9
ICT1	(2,3)	(3,4)	(3,4)	(2,3)	(4,5)	(2,3)	(4,5)	(3,4)	(4,5)
ICT2	(4,5)	(4,5)	(4,5)	(0,1)	(4,5)	(4,5)	(3,4)	(4,5)	(3,4)
ICT3	(4,5)	(4,5)	(4,5)	(1,2)	(1,2)	(2,3)	(3,4)	(2,3)	(1,2)
ICT4	(3,4)	(3,4)	(3,4)	(4,5)	(3,4)	(1,2)	(3,4)	(2,3)	(3,4)
ICT5	(3,4)	(3,4)	(4,5)	(4,5)	(3,4)	(2,3)	(3,4)	(3,4)	(4,5)
ICT^	(4,5)	(2,3)	(3,4)	(4,5)	(3,4)	(1,2)	(2,3)	(1,2)	(0,1)
ICT7	(3,4)	(3,4)	(4,5)	(3,4)	(3,4)	(4,5)	(3,4)	(4,5)	(2,3)
ICT8	(2,3)	(4,5)	(3,4)	(2,3)	(2,3)	(0,1)	(2,3)	(0,1)	(1,2)
ICT9	(3,4)	(4,5)	(3,4)	(0,1)	(4,5)	(3,4)	(3,4)	(3,4)	(0,1)
ICT10	(4,5)	(3,4)	(4,5)	(0,1)	(4,5)	(4,5)	(4,5)	(4,5)	(2,3)
ICT11	(2,3)	(3,4)	(3,4)	(4,5)	(3,4)	(2,3)	(4,5)	(3,4)	(2,3)
ICT12	(2,3)	(2,3)	(3,4)	(3,4)	(2,3)	(3,4)	(4,5)	(3,4)	(0,1)
ICT13	(4,5)	(3,4)	(4,5)	(2,3)	(2,3)	(4,5)	(3,4)	(4,5)	(0,1)
ICT14	(2,3)	(2,3)	(4,5)	(4,5)	(2,3)	(3,4)	(4,5)	(0,1)	(3,4)

To further analyze and prioritize these obstacles, the study calculated the overall grey weight using Eq. (1) and subsequently converted it into a crisp number using Eq. (2). The precise numerical data were instrumental in determining whether to include or exclude obstacles from this subsequent study. Obstacles with a crisp value exceeding 3.5 were included, while those below this threshold were excluded^82,83^. Detailed measurements of both grey and crisp weight, along with corresponding decisions, are provided in Table 7. This table provides a comprehensive overview of the outcomes of the Grey Delphi method, aiding in the identification and classification of critical barriers with a strategic decision-making process. The decisions to either select or reject each obstacle, along with their respective crisp weights, guide the subsequent steps in addressing and mitigating the barriers to the adoption of blockchain technology in the Chinese Fisheries Supply Chain.

**Table 7:** Results of the grey Delphi method.

Initial barrier	Overall grey weight	Crisp weight	Decision	Rename
ICT1	(3.0,4.0)	3.5	Select	BBT1
ICT2	(3.3,4.3)	3.8	Select	BBT2
ICT3	(2.4,3.4)	2.9	Reject	NA
ICT4	(2.7,3.7)	3.2	Reject	NA
ICT5	(3.2,4.2)	3.7	Select	BBT3
ICT^	(2.2,3.2)	2.7	Reject	NA
ICT7	(3.2,4.2)	3.7	Select	BBT4
ICT8	(1.7,2.7)	2.2	Reject	NA
ICT9	(2.5,3.5)	3	Reject	NA
ICT10	(3.2,4.2)	3.8	Select	BBT5
ICT11	(2.8,3.8)	3.3	Reject	NA
ICT12	(2.4,3.4)	2.9	Reject	NA
ICT13	(2.8,3.8)	3.3	Reject	NA
ICT14	(2.6,3.6)	3.1	Reject	NA

In this way, the work identified five significant obstacles associated with the adoption of blockchain technology in emerging economies. Additionally, the confirmed barriers to embracing blockchain technology are detailed in Table 8. These barriers highlight the multifaceted challenges faced by the fisheries industry in adopting blockchain technology, ranging from logistical complexities to financial constraints and regulatory intricacies. The detailed descriptions provide a nuanced understanding of each identified obstacle, forming a foundation for targeted strategies and interventions to enhance blockchain adoption in the Fisheries Supply Chain. Additionally, Fig. 2 visually represents the confirmed barriers, offering a graphical depiction of their significance and interrelationships.

**Table 8:** Finalized barriers of blockchain technologies in FSC with description.

S.no	Code	Barrier	Description
1	BBT1	Complex supply chain network	Fisheries supply chains can be highly complex, involving numerous intermediaries, from fishermen to processors to distributors. Implementing a blockchain system that accurately captures and tracks every step of the supply chain can be challenging
2	BBT2	Data accuracy and trust	Blockchain relies on the accuracy of the data entered into the system. Ensuring that the data about the origin, quality, and handling of seafood is accurate and trustworthy is essential to maintain the integrity of the blockchain
3	BBT3	Cost of implementation	Implementing a blockchain system, including hardware, software, and training, can be expensive. Small and medium-sized fisheries enterprises may face financial barriers to adoption
4	BBT4	Regulatory compliance	The seafood industry is subject to various regulations related to food safety, sustainability, and traceability. Ensuring that a blockchain system complies with these regulations is crucial but can be complicated
5	BBT5	Privacy and data security	While blockchain offers transparency, it must also protect sensitive information. Ensuring that personal or proprietary data is secure can be challenging

#### DEMATEL analysis

The Decision-Making Trial and Evaluation Laboratory (DEMATEL) is recognized as a valuable approach for pinpointing the cause-and-effect relationships within intricate systems. DEMATEL enables the assessment of interlinked factors and the identification of critical elements by constructing a visual structural model^84^. DEMATEL analysis is a powerful technique for understanding and managing complex systems, and it has been applied in various fields to solve problems and improve decision-making processes. The study conducted an analysis using grey DEMATEL to explore the causal relationships among Critical Barrier Factors (CBFs) in the adoption of blockchain technology in FSC. To guide the experts through the research process, we initially outlined the study's objectives. The experts were then asked to assess how one CBF influences another, using a linguistic scale in the form of a direct-relationship matrix. We converted the original direct relationship matrices into grey relationship matrices using grey numbers. With input from six experts, we obtained six grey initial relationship matrices, which were subsequently combined to form a global grey relation matrix presented in Table 9.

**Table 9:** Initial relationship matrix of blockchain technologies in FSC.

Barriers	BBT1	BBT2	BBT3	BBT4	BBT5
BBT1	0	2.125	3.0125	1.625	2.225
BBT2	3.375	0	1.875	3.25	3.375
BBT3	2.625	2.25	0	2.65	1.875
BBT4	3.5	3.5	2.625	0	3.75
BBT5	1.375	3.125	3.375	2.75	0

Initially, the expert panel provided the Initial Direct Relationship Matrix (IDRM). Among these experts, six possess expertise in fisheries supply chain management, technology adoption, and Blockchain. Using Eq. (1), overall direct relationship matrix was created, which is presented in Table 5. Subsequently, we transformed the IDRM into a Normalized Relationship Matrix (NRM) using Eqs. (2) and (3), and the resulting NRM is displayed in Table 10. Equation (4) was used to convert the obtained Normalized Relationship Matrix (NRM) into a total relationship matrix (T), and the resulting matrix is displayed in Table 11.

**Table 10:** Normalized relationship matrix.

Barriers	BBT1	BBT2	BBT3	BBT4	BBT5
BBT1	0	0.159	0.225	0.121	0.166
BBT2	0.252	0	0.140	0.243	0.252
BBT3	0.196	0.168	0	0.198	0.140
BBT4	0.262	0.262	0.196	0	0.280
BBT5	0.103	0.234	0.252	0.206	0

**Table 11:** Total relationship matrix.

Barriers	BBT1	BBT2	BBT3	BBT4	BBT5
BBT1	0.601	0.745	0.792	0.688	0.758
BBT2	0.980	0.791	0.911	0.942	1.007
BBT3	0.804	0.789	0.640	0.775	0.780
BBT4	1.062	1.075	1.025	0.820	1.102
BBT5	0.815	0.915	0.919	0.860	0.736

**Figure 3:**
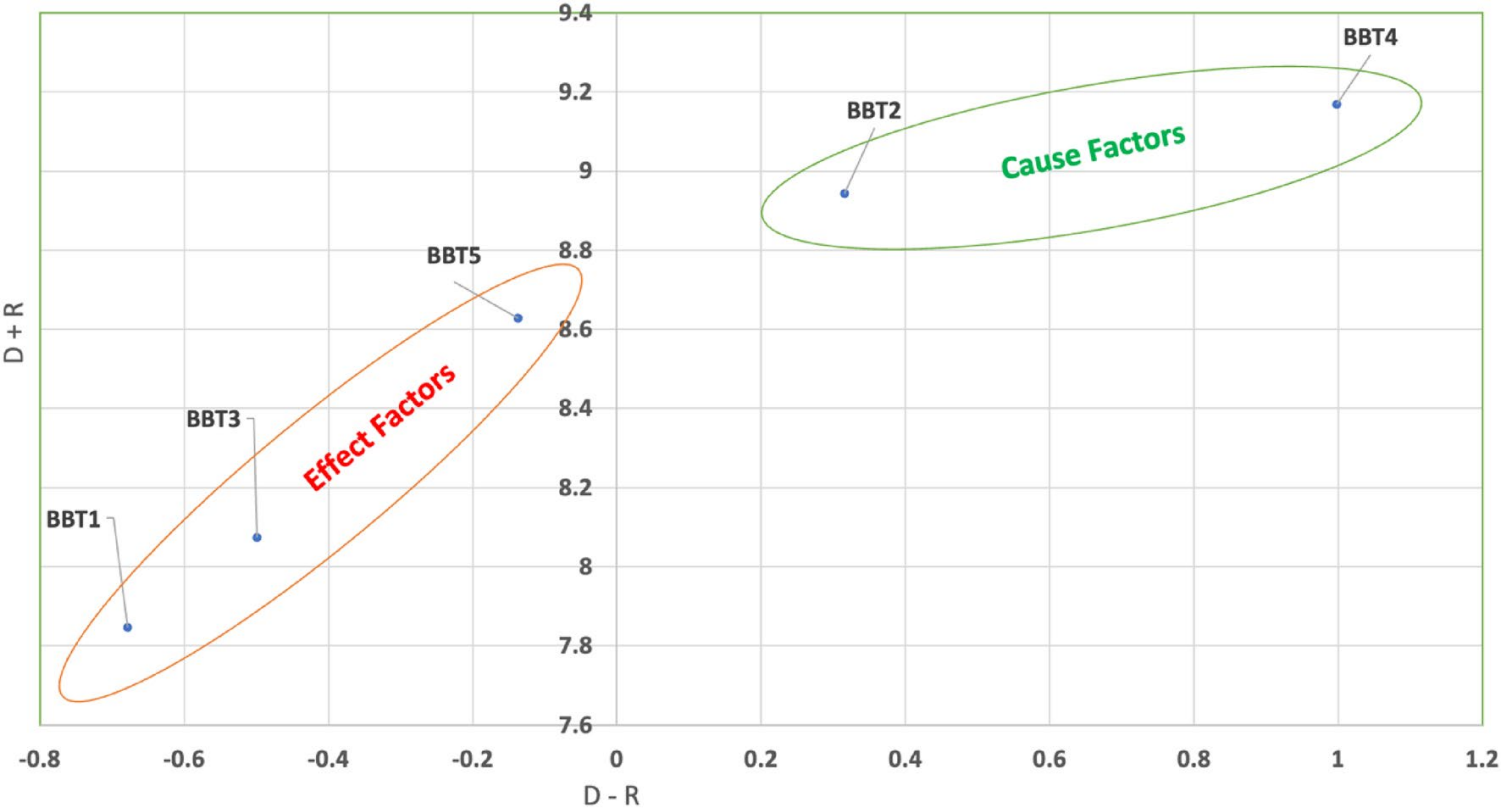
Causal relationship map among barriers of BT adoption in the fisheries supply chain.

The work calculated a threshold value to identify significant relationships among the barriers. This threshold is determined by adding the average and standard deviation of the T matrix. It helps differentiate the structure and aids in developing the causal map. If the values in Table 11's T matrix exceed this threshold, a causal map is drawn. This cause-and-effect map not only helps assess the importance of one barrier over another but also filters out minor effects. The causal map for blockchain technology adoption barriers, constructed using the T matrix in Table 11, is shown in Fig. 3. Additionally, the map distinguishes cause-and-effect barriers with different colors.

Later the row-wise summation (R) and column-wise summation (C) in the total relationship matrix T were calculated using Eqs. (5) and (6), which are displayed in Table 12. Additionally, the prominence scores and effect scores with the assistance of Eqs. (7) and (8), respectively were determined.

**Table 12:** Causal relationships among barriers to blockchain technology adoption in the fisheries supply chain.

	D	R	D + R	D − R	Cause/effect
BBT1	3.584	4.262	7.847	− 0.678	Effect
BBT2	4.630	4.314	8.943	0.316	Cause
BBT3	3.787	4.286	8.074	− 0.499	Effect
BBT4	5.083	4.085	9.168	0.998	Cause
BBT5	4.245	4.383	8.628	− 0.138	Effect

**Figure 4:**
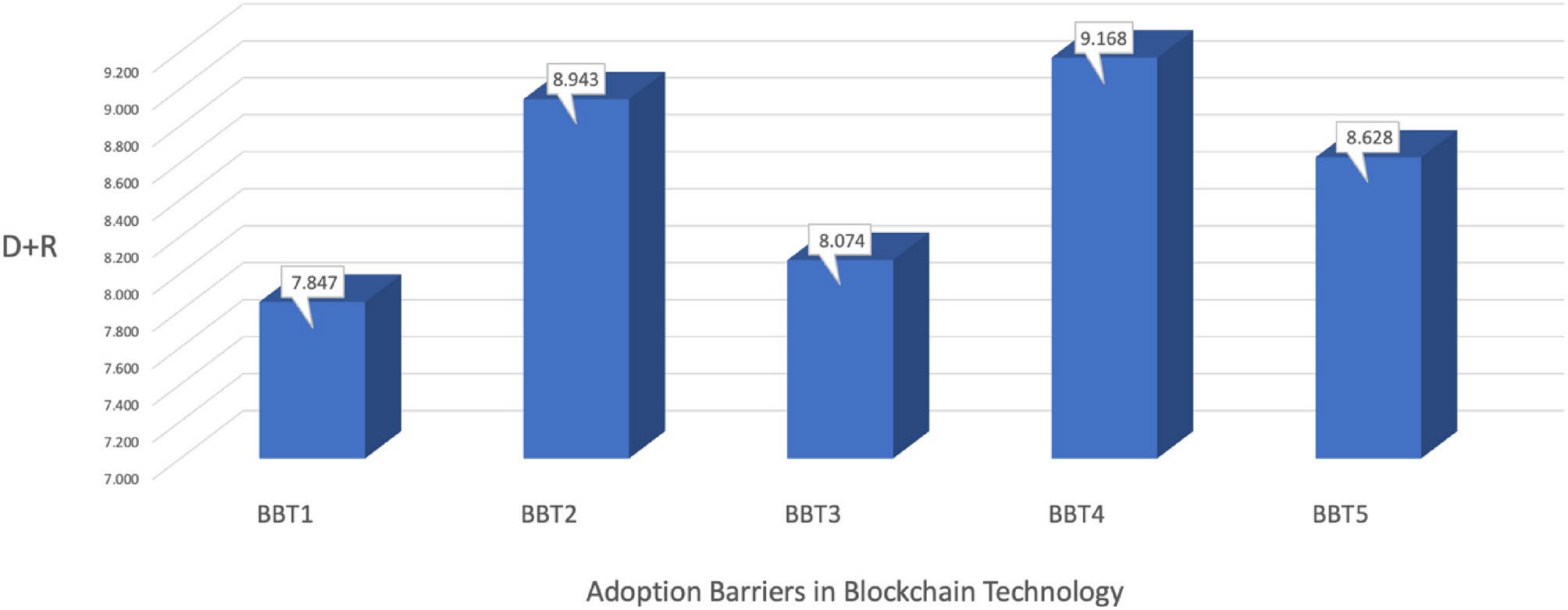
Ranking of the barriers to BT implementation in the fisheries supply chain.

The DEMATEL method was used to determine the order of importance for each barrier. The degree of influence between the identified constructs is indicated by the relationship intensity, while (D—R) signifies the criteria for the cause-and-effect grouping. However, the central strength of these constructs is represented by the (D + R) value. Typically, the cause group construct operates independently and propels the effect group construct, as observed in the study by^85^. The critical order of the barriers is as follows: ' Regulatory compliance ' is ranked highest, followed by 'Data accuracy and trust,' ‘Privacy and data security,' 'Cost of implementation,' and finally, ' Complex supply chain network.' The importance order of each barrier is illustrated in Fig. 4.

### Sensitivity analysis

Conducting a sensitivity analysis involves assessing the robustness of results. In this context, the approach employed includes altering the weighting assigned to one expert's input while maintaining uniform weightings for the remaining experts. Various methods can be employed for this purpose, such as adjusting the weight levels attributed to individual experts or different barriers. In this study, archetypal sensitivity analysis is utilized, wherein distinct weightings are assigned to experts. To illustrate, initially, the weight assigned to Expert 1 was set at 0.2, while the other experts were given a weight of 0.1. To conduct sensitivity analysis, nine distinct total relationship matrices were generated by multiplying the weights assigned to various experts as outlined in Table 6 and similar matrices. Subsequently, average relationship matrices were calculated, leading to the establishment of cause-effect relationships among different barriers. The weights assigned to the nine experts and the ranking of diverse barriers during the sensitivity analysis are presented in Tables 13 and 14. From the table, it is clear that there was no major change in barrier rankings after sensitivity analysis. The same rank order for cause-effect barriers for each expert was obtained, accepting minor rank value variation. Therefore, the sensitivity analysis confirms the robustness of obtained results.

**Table 13:** Weight given to nine experts for sensitivity analysis.

	E1	E2	E3	E4	E5	E6	E7	E8	E9
Scenario 1	0.2	0.1	0.1	0.1	0.1	0.1	0.1	0.1	0.1
Scenario 2	0.1	0.2	0.1	0.1	0.1	0.1	0.1	0.1	0.1
Scenario 3	0.1	0.1	0.2	0.1	0.1	0.1	0.1	0.1	0.1
Scenario 4	0.1	0.1	0.1	0.2	0.1	0.1	0.1	0.1	0.1
Scenario 5	0.1	0.1	0.1	0.1	0.2	0.1	0.1	0.1	0.1
Scenario 6	0.1	0.1	0.1	0.1	0.1	0.2	0.1	0.1	0.1
Scenario 7	0.1	0.1	0.1	0.1	0.1	0.1	0.2	0.1	0.1
Scenario 8	0.1	0.1	0.1	0.1	0.1	0.1	0.1	0.2	0.1
Scenario 9	0.1	0.1	0.1	0.1	0.1	0.1	0.1	0.1	0.2

**Table 14:** Comparative ranking of cause/effect relationships among common barriers obtained from sensitivity analysis.

Barrier factor	Barrier code	DEMATEL (D–R)	Sensitivity analysis (D–R)	Rank
Regulatory compliance	BBT4	− 0.678	0.280	1
Data accuracy and trust	BBT2	0.316	0.088	2
Privacy and data security:	BBT5	− 0.499	− 0.039	3
Cost of implementation	BBT3	0.998	− 0.140	4
Complex supply chain network	BBT1	− 0.138	− 0.190	5

## Discussion

The complexity of the fisheries supply chain emerged as a prominent barrier, given the involvement of numerous intermediaries. To overcome this, it is crucial to comprehensively map the supply chain network, streamline processes, establish interoperability standards, select scalable blockchain solutions, and implement permissioned networks. Ensuring data accuracy and trust is essential, and mechanisms for data validation and verification within the blockchain should be implemented. Additionally, strategies such as robust supplier onboarding procedures and secure authentication mechanisms can enhance data accuracy and trust. Implementing blockchain technology involves various costs, posing a financial barrier, especially for small and medium-sized fisheries enterprises. To address this challenge, a comprehensive cost–benefit analysis is crucial, and a phased implementation approach, collaboration with stakeholders, exploration of open-source blockchain solutions, and seeking government grants and incentives can help alleviate the financial burden. Regulatory compliance is a significant barrier in the seafood industry, subject to diverse and evolving regulations. Open communication with regulatory authorities, integration of compliance features into blockchain design, and leveraging smart contracts for automated compliance checks are recommended strategies. Robust data privacy and security measures, comprehensive auditing, and reporting tools are crucial for compliance. While blockchain offers transparency, protecting sensitive information is equally important, necessitating strong data privacy and security measures. The DEMATEL analysis revealed causal relationships among the identified barriers, with 'Regulatory Compliance' emerging as the most influential barrier, followed by 'Cost of Implementation' and 'Complex Supply Chain Network.' Overcoming these barriers requires a multifaceted approach considering technology, compliance, financial aspects, and data security, and collaboration among stakeholders, ongoing monitoring, and adaptation to changing regulations are instrumental in addressing these challenges, offering valuable insights for industry players, policymakers, and researchers seeking to enhance transparency, traceability, and sustainability within the fisheries supply chain through blockchain technology.

However, China faces specific barriers that are influenced by its unique socio-economic and regulatory environment. The country's complex supply chain network (BBCT1) is a distinctive challenge due to the sheer scale and diversity of its fisheries industry. This complexity can hinder the seamless integration of blockchain across the entire supply chain^61^. Additionally, regulatory compliance (BBCT7) is a significant concern in China, given the government's active involvement in shaping the technology landscape^86^. China's regulatory framework may require adjustments and alignment with blockchain technology adoption.

On the other hand, China's comparatively strong emphasis on technology development and innovation might serve as an advantage in addressing these barriers. China's rapid progress in technology adoption and its large market size can potentially accelerate the resolution of these challenges^65^. Nevertheless, it remains essential for both China and the global fisheries industry to address these shared and unique barriers effectively to harness the full potential of blockchain technology for enhanced sustainability and food security in the fisheries supply chain.

### Effect CBF’s

The study categorized the identified barriers as 'effect barriers' and 'cause barriers.' The group of 'effect barriers' comprises three key barriers: 'Regulatory Compliance,' 'Cost of Implementation,' and 'Complex Supply Chain Network.' These barriers demand greater attention, as they exert influence on other significant barriers. The most influential barrier is regulatory compliance. Regulatory compliance serves as a significant obstacle to the widespread adoption of blockchain technology within the fisheries supply chain. This barrier arises from the complex and dynamic nature of regulations governing the fishing industry, which vary across regions and evolve over time. Blockchain implementation in this context requires alignment with diverse legal frameworks, making it a complex and resource-intensive endeavor. Additionally, ensuring transparency and traceability in compliance with these regulations through blockchain technology can be challenging, further impeding its adoption. Addressing these regulatory hurdles is crucial for realizing the full potential of blockchain in enhancing transparency and sustainability within the fisheries supply chain.

Overcoming the barrier of regulatory compliance in blockchain adoption within the fisheries supply chain entails a multifaceted approach. This involves establishing open lines of communication with regulatory bodies and industry stakeholders to align blockchain strategies with existing regulations. Leveraging expertise in fisheries regulations for tailored solutions, developing adaptable blockchain systems to accommodate evolving compliance requirements, implementing robust data security measures, and utilizing smart contracts for automated compliance checks are crucial steps. Furthermore, maintaining comprehensive audit trails, educating stakeholders, conducting pilot programs to showcase benefits, engaging in advocacy efforts for regulatory reforms, and continuously monitoring and adapting blockchain solutions to changing regulations are essential components of this comprehensive approach.

The next effect barriers is cost of implementation. To overcome this barrier in the fisheries supply chain, a strategic approach is essential. This involves conducting a comprehensive cost–benefit analysis to highlight blockchain's potential to enhance transparency, reduce fraud, improve traceability, and enable better decision-making in the supply chain. Implementing a phased approach, starting with smaller-scale pilot projects, allows for a thorough assessment of costs and benefits before full-scale deployment. Collaboration with supply chain stakeholders can distribute financial burdens, while exploring open source blockchain solutions and leveraging third-party providers can reduce expenses. Investigating government grants and incentives, adopting lean development practices, investing in staff training, and continuously monitoring ROI help ensure that the benefits of blockchain outweigh initial implementation costs.

The final effect barrier is complex supply chain network and to overcome this barrier it begins with mapping the entire network comprehensively, encompassing stakeholders, processes, and data flows to gain a clear understanding of the complexity. Identifying opportunities to simplify or streamline processes helps reduce unnecessary intricacies. The establishment of interoperability standards ensures seamless communication between various systems and the blockchain, mitigating integration challenges. Choosing a scalable blockchain solution accommodates network growth, while implementing permissioned networks enhances control and security. Ensuring data consistency and standardization, along with education and training, fosters effective adaptation. Pilot projects validate compatibility and effectiveness, expert consultation tailors solutions, and continuous improvement and collaboration across stakeholders collectively address the intricacies of the supply chain, promoting successful blockchain adoption.

### Cause CBF’s

The cause barrier group includes ‘Regulatory Compliance’ and ‘Data Accuracy and Trust’. Amongst the two, the most cause barrier is regulatory compliance which can be overcome by considering a multifaceted approach. Engage in open communication with regulatory authorities to understand their specific requirements. Leverage regulatory expertise by consulting specialists in fisheries regulations and integrate compliance into the blockchain's design. Utilize smart contracts to automate and monitor compliance and implement robust data privacy and security measures. Integrate auditing tools, explore regulatory sandboxes, and collaborate with stakeholders. Stay updated on evolving regulations, educate your team, maintain transparency with regulators, and engage in advocacy efforts for blockchain-friendly reforms. These strategies collectively enable compliance while harnessing blockchain's potential for transparency and traceability. In order to overcome the data accuracy and trust there is a need to engage in open communication with regulatory authorities to understand their specific requirements^62^. Leverage regulatory expertise by consulting specialists in fisheries regulations and integrate compliance into the blockchain's design^31^. Utilize smart contracts to automate and monitor compliance and implement robust data privacy and security measures. Integrate auditing tools, explore regulatory sandboxes, and collaborate with stakeholders. Stay updated on evolving regulations, educate your team, maintain transparency with regulators, and engage in advocacy efforts for blockchain-friendly reforms. These strategies collectively enable compliance while harnessing blockchain's potential for transparency and traceability.

## Managerial and policy implications

The findings of our research carry significant implications for decision-makers engaged in the implementation of Sustainable Fisheries Supply Chain Management (SFSCM). The study has yielded several managerial recommendations. The impact group can be readily influenced by the cause group, underscoring the importance of managers directing their attention primarily towards addressing causal barriers when integrating SFSCM practices into their conventional supply chains. This research aids managers in delineating barriers that demand heightened focus within their respective industries, distinguishing between those of greater and lesser significance. The hierarchical classification of barriers in the cause and effect groups provides valuable assistance to managers and decision-makers in formulating strategic policies during the implementation of SSCM. The outcomes of this research framework have the potential to motivate decision-makers and industrial managers to embrace SFSCM practices that are pivotal for sustainable development and exert the most substantial influence on the transformation of traditional supply chains in the fisheries sector. Managers can view this framework as a benchmark for enhancing traditional supply chains, thereby fostering improvements in environmental, social, and economic sustainability.

Based on the findings of the research on blockchain technology barriers in fisheries supply chains, several key policy implications emerge. Government entities should focus on nurturing trust within the industry by facilitating industry group discussions and educational programs to enhance awareness and understanding of blockchain technology (BT). Moreover, improving information infrastructure, including data security standards, can alleviate concerns regarding privacy and data security. Policymakers can incentivize BT adoption through tax benefits and grants, foster partnerships with technology providers to address technical hurdles, and enforce transparency and accountability measures within the supply chain. Cost mitigation options, particularly for small and medium-sized enterprises (SMEs), and the promotion of a culture of information sharing can further reduce adoption barriers. Implementing performance metrics and supporting research and development initiatives are essential steps in creating an enabling environment for successful BT adoption, ultimately enhancing transparency, efficiency, and sustainability in the fisheries sector.

The work also has significant policy implications for businesses and industry leaders. To overcome primary barriers such as information sharing readiness and trust issues, executives can invest in relationship-building efforts, align incentives, and consider co-investing in blockchain technology (BT) initiatives. Leveraging third-party technologies and training programs can help businesses source expertise and address these hurdles effectively. Furthermore, formal business case evaluations of BT adoption, articulating its net benefits, can encourage companies to shift their information policies and corporate culture towards more openness and collaboration. These findings offer valuable insights for policymakers and business leaders to proactively anticipate and mitigate potential implementation challenges, fostering a climate of innovation and cooperation within fisheries supply chains. This approach can ultimately lead to improved data and knowledge sharing, creating win–win outcomes across supply chains, enhancing food security, and promoting sustainability in the industry.

## Conclusion and future work

The comprehensive exploration of challenges and barriers associated with implementing blockchain technology in fisheries supply chains reveals a complex landscape that demands nuanced strategies for successful integration. Drawing upon an extensive literature review and utilizing the Scopus database, this study employed the grey Delphi method, with input from both academic and industry experts, to identify fourteen Critical Barrier Factors (CBFs). These factors represent key obstacles hindering the effective assimilation of blockchain technology into supply chains, forming the foundation for further analysis. The study sheds light on five significant obstacles specifically linked to the adoption of blockchain technology in emerging economies within the fisheries industry. These obstacles underscore the multifaceted challenges faced by fisheries, encompassing logistical complexities, financial constraints, and regulatory intricacies. The Decision-Making Trial and Evaluation Laboratory (DEMATEL) method, employed to explore causal relationships among CBFs, generated valuable insights. Regulatory compliance emerged as the most pivotal, followed by data accuracy and trust, privacy and data security, the cost of implementation, and the complexity of the supply chain network. DEMATEL not only identifies critical barriers but also unravels their intricate relationships. This nuanced understanding provides actionable guidance for policymakers, industry practitioners, and researchers, facilitating the formulation of targeted interventions and strategies crucial for the successful adoption of blockchain technology in fisheries supply chains.

Regulatory compliance poses a formidable challenge, given the diverse and evolving nature of regulations in the seafood industry. Recommended strategies include open communication with regulatory authorities, integration of compliance features into blockchain design, and the use of smart contracts for automated compliance checks. Robust data privacy and security measures, comprehensive auditing, and reporting tools are emphasized for achieving compliance. The unique socio-economic and regulatory environment of China introduces specific barriers, necessitating tailored approaches to enable seamless integration of blockchain across the entire supply chain. The study categorizes barriers as 'efffect' and 'cause,' with regulatory compliance identified as the most influential due to the dynamic nature of fishing industry regulations. Addressing regulatory hurdles is crucial for unlocking the full potential of blockchain in enhancing transparency and sustainability within fisheries supply chains.

To overcome the challenges associated with the complex supply chain network, the study recommends a collaborative approach involving comprehensive mapping, simplification of processes, establishment of interoperability standards, selection of scalable blockchain solutions, implementation of permissioned networks, ensuring data consistency and standardization, and continuous improvement through stakeholder collaboration. The research highlights regulatory compliance and data accuracy and trust as the most cause barriers in the implementation of Sustainable Fisheries Supply Chain Management (SFSCM). To address these, managers are advised to engage in open communication with regulatory authorities, leverage regulatory expertise, integrate compliance into blockchain design, use smart contracts, employ auditing tools, explore regulatory sandboxes, and collaborate with stakeholders. Staying updated on evolving regulations, maintaining transparency, and advocating for blockchain-friendly reforms are also recommended strategies.

The study, while comprehensive in its examination of blockchain technology (BT) adoption barriers within the fisheries supply chain, is not without its limitations. Firstly, the barriers were initially identified and refined based on a combination of literature survey and expert opinions. Although extensive efforts were made to encompass a wide spectrum of perspectives, it is possible that certain potential barriers may have been inadvertently overlooked in the selection and review process. Additionally, the evaluation heavily relies on expert feedback, which, despite the study’s design efforts to minimize bias, may still introduce some degree of subjectivity.

To mitigate these limitations, future research endeavors should consider broader validation and refinement processes to enhance this collective understanding of BT barriers. Firstly, researchers should delve into the dynamic nature of regulatory compliance, given its influential role in obstructing the adoption of blockchain technology in the fisheries supply chain. A more in-depth investigation into the evolving regulatory landscape, both globally and regionally, could provide a nuanced understanding of the challenges posed and contribute to the development of adaptive strategies. This could involve exploring the feasibility and effectiveness of technological solutions, such as smart contracts, in automating compliance checks and adapting to regulatory changes in real-time. Secondly, future work should focus on refining and expanding the strategies proposed for overcoming the financial barriers associated with implementing blockchain technology, particularly for small and medium-sized fisheries enterprises. Further research can investigate the long-term cost-effectiveness of different blockchain solutions, the scalability of open-source options, and the potential impact of government grants and incentives. Additionally, exploring innovative financing models and collaborations within the industry could contribute to more sustainable and feasible financial approaches. Addressing the unique challenges faced by the fisheries industry in emerging economies, especially in China, requires specific attention in future research. Tailored strategies for navigating the socio-economic and regulatory landscape of these regions can be developed to facilitate the seamless integration of blockchain technology across the entire supply chain. Comparative studies between emerging and developed economies could provide valuable insights into the contextual variations influencing blockchain adoption.

## Data Availability

The datasets used and analyzed during the current study are available from the corresponding author on reasonable request.
